# Evidence for the need for vision screening of school children in Turkey

**DOI:** 10.1186/s12886-017-0618-9

**Published:** 2017-12-02

**Authors:** Serap Azizoğlu, Sheila G. Crewther, Funda Şerefhan, Ayla Barutchu, Sinan Göker, Barbara M. Junghans

**Affiliations:** 1 School of Psychology and Public Health, La Trobe University, Bundoora, VIC 3086 Australia; 2 Istanbul Surgery Hospital, Şişli, 34365 Istanbul, Turkey; 3 Optometry, School of Medicine, Deakin University, Geelong, VIC 3220 Australia; 4 Department of Experimental Psychology, University of Oxford, Oxford, OX1 3UD UK; 5 School of Optometry and Vision Science, University of New South Wales Australia, UNSW, Sydney, 2052 Australia

**Keywords:** Public health, Socioeconomic status, Primary school children, Middle East, Visual screening, Refractive errors

## Abstract

**Background:**

In many countries, access to general health and eye care is related to an individual’s socioeconomic status (SES). We aimed to examine the prevalence of oculo-visual disorders in children in Istanbul Turkey, drawn from schools at SES extremes but geographically nearby.

**Methods:**

Three school-based vision screenings (presenting distance visual acuity, cover test, eye assessment history, colour vision, gross stereopsis and non-cycloplegic autorefraction) were conducted on 81% of a potential 1014 primary-school children aged 4–10 years from two private (high SES) schools and a nearby government (low SES) school in central Istanbul. Prevalence of refractive errors and school-based differences were analysed using parametric statistics (ANOVA). The remaining oculo-visual aspects were compared using non-parametric tests.

**Results:**

Of the 823 children with mean age 6.7 ± 2.2 years, approximately 10% were referred for a full eye examination (8.2% and 16.3% of private/government schools respectively). Vision had not been previously examined in nearly 22% of private school children and 65% of government school children. Of all children, 94.5% were able to accurately identify the 6/9.5 [LogMAR 0.2] line of letters/shapes with each eye and 86.6% the 6/6 line [LogMAR 0], while 7.9% presented wearing spectacles, 3.8% had impaired colour vision, 1.5% had grossly impaired stereo-vision, 1.5% exhibited strabismus, 1.8% were suspected to have amblyopia and 0.5% had reduced acuity of likely organic origin. Of the 804 without strabismus, amblyopia or organic conditions, 6.0% were myopic ≤ − 0.50DS, 0.6% hyperopic ≥ + 2.00DS, 7.7% astigmatic ≥1.00 DC and 6.2% anisometropic ≥1.00DS.

**Conclusions:**

The results highlight the need for general vision screenings for all children prior to school entry given the varied and different pattern of visual problems associated with lifestyle differences in two populations raised in the same urban locale but drawn from different socioeconomic backgrounds.

**Electronic supplementary material:**

The online version of this article (10.1186/s12886-017-0618-9) contains supplementary material, which is available to authorized users.

## Background

In many countries, access to general health and eye care is related to an individual’s socioeconomic status (SES), with lower SES individuals more likely to experience visual impairment [1] due to limited routine preventive care on a timely basis [2–4]. One way to detect and manage vision anomalies would be for every child to visit an eye care practitioner regularly, though, the cost is out of reach for many socio-economic groups [2–4] especially in countries where there are limited numbers of practitioners available to carry out full eye examinations [5]. An alternative is public health screening programs aimed at detecting and referring those children with significant issues that may lead to functional and preventable blindness [6] or that may potentially perturb the educational horizons of the child [7]. The costs and effects of screening school children for refractive errors has been well-reviewed recently for different WHO sub-regions in Africa, Asia, America and Europe and the long term economic benefits established [8]. However, a cost analysis has not been reported for Turkey and the neighbouring Middle East.

Currently, prevalence data covering refractive errors, amblyopia or other vision anomalies such as strabismus are not available for children living in the largest urban population in Turkey, namely Istanbul. However, a number of studies of children from what is often considered to be the lower socioeconomic groups in more rural areas of Eastern Turkey do exist [9–12] (see Table 1). In general these rural studies have reported a low prevalence of oculo-visual disorders [9–12], although in 2013 Gursoy et al. found a significantly higher prevalence of myopia leading them to call for a large-scale national screening program [10].

**Table 1:** Prevalence of refractive errors^a^, amblyopia and strabismus findings from studies across Turkey and surrounding countries

Location	Authors	Comments	Myopia	Hyperopia	Astigmatism	Strabismus	Amblyopia	Presented with spectacles	How many referred	Visual acuity
Istanbul, Turkey, central suburb	Current study, 2009	4–10 years *N* = 823 Extremes of SES Non-cycloplegic	6.0% ≤ −0.50DS	0.6% ≥ +2.00DS	7.7% ≥1.00 DC 12.9% ≥0.75D	1.5%	1.8%	7.9%	10.4%	2.1% ≥ 6/12 habitual in better eye
Ankara, Turkey, capital city and nearby villages	Turacli, Aktan & Duruk. 1995 [82]	Primary schools N = 23,810 Range of SES, private/state schools. Cycloplegia not stated	3.52% ≤ −0.50DS	2.31% > + 0.75D	5.17% ≥0.75 DC	2.5%	1.1%	–	–	–
Elazığ rural eastern Turkey	Polat & Akyol. 2003 [11]	7–9 yrs. *N* = 234 78% low SES Cycloplegia not stated	3.2% degree not stated	8.7% degree not stated	–	2.8%	3.2%	–	5.5%	5.5% ≥ 6/30 habitual in better eye
Sivrihisar, rural western Turkey	Unsal, Ayranci, Tozun. 2009 [12]	6–7 yrs. Mean age 10.5 ± 2.3 *N* = 1606 Cycloplegic	N/A	N/A	N/A	1.7%	5.0%	8.6%	–	1.7% ≥ 6/12 habitual in better eye
Malatya, rural eastern Turkey	Cumurcu et al. 2011 [35]	7–15 yrs. *N* = 661 Cycloplegic	5.59% degree not stated	2.87% degree not stated	41.5% degree not stated	3.02%	1.21%	–	–	–
Kirrikale, rural eastern Turkey	Ergin. 2011 [36]	Primary schools *N* = 2386 Cycloplegic	4.55% degree not stated	16.30% degree not stated	79% degree not stated	2.43	1.2%	1.7%	21.6% (some type of refractive error and/or ocular pathology)	–
Eskisehir, eastern Turkey, university town of 650,000	Gursoy et al. 2013 [10]	7–8 yrs. *N* = 721 Cycloplegic	22.6% ≤ −0.50DS	10.6% > +0.75DS	11.0% ≥0.75 DC	2.5%	5.5%	9.0%	9.0%	7.8% ≥ 6/7.5 best corrected in better eye
Diyarbakir, rural eastern Turkey	Caca et al. 2013 [9]	6–14 yrs. Mean 10.6 ± 3.6 N = 21,062 Cycloplegic	3.2% ≤ −0.50DS	5.9% ≥ +2.00DS	14.3% ≥0.50 DC	2.4%	2.6%	12.1%	–	1/7% ≥ 6/12 habitual in better eye
Istanbul, Turkey	Onal et al. 2007 [51]	*N* = 207 medical students Mean age 21.1 ± 1.6 yrs. Cycloplegic	32.9% ≤ −0.75D	–	–	–	–	–	–	–
Stara Zagora, Bulgaria, suburb of large rural city	Plainis et al. 2009 [83]	10–15 yrs. *N* = 310 Non-cycloplegic	14.1% ≤ −0.75DS + VA at least 6/7.5 primary school	Not stated	9.7% >0.75 DC in at least one eye	–	–	8.7%	–	5.2% ≥ 6/19 habitual in better eye
Heraklion, Greece, large rural city	Plainis et al., 2009 [83]	10–15 yrs. *N* = 588 Non-cycloplegic	28.9% ≤ −0.75DS + VA at least 6/7.5	Not stated	16.8% >0.75 DC in at least one eye	–	–	23.3%	–	11.7% ≥ 6/19 habitual in better eye
Shahrood, Iran, rural and urban	Jamali et al. 2009 [71]	6 yrs. *N* = 827 Cycloplegic	1.7% ≤ −0.50DS	20.5% ≥ +2.00DS	19.6% ≥0.75 DC	1.2%	1.7%	3.5%	–	0.2% >6/12 best corrected in better eye
Dezful, Iran, rural and urban	Fotouhi et al. 2007 [70]	Grades 1–12 Mean age 11.0 yrs. *N* = 5544 Cycloplegic	3.4% ≤ −0.50DS	16.6% ≥ +2.00DS	18.7% ≥0.75 DC	0.8%	–	–	–	0.3% >6/12 best corrected in better eye
Bojnourd, Iran, rural	Rezvan et al. 2012 [73]	Age 7-15 yrs. *N* = 1551 Mean age 11.2 ± 2.4 yrs. Cycloplegic	4.3% ≤ −0.50DS	5.4% ≥ +2.00DS	11.5% ≥0.75 DC	–	–	–	–	0.2% >6/12 best corrected in better eye
Shiraz, Iran, rural city	Yekta et al. 2010 [59]	Grades 1–8 *N* = 2130 Mean age 11.2 ± 2.4 yrs. Cycloplegic	4.4% ≤ −0.50DS	5.9% ≥ +2.00DS	11.3% ≥0.75 DC	–	–	–	–	0.9% >6/12 best corrected in better eye
Masshad, Iran, large rural centre	Ostadimoghaddam et al. [84]	Ages 7–14 *N* = 1163 Cycloplegic	2.4% ≤ −0.50DS	2.5% ≥ +2.00DS	9.8% ≥0.75 DC	–	–	–	–	–

^a^Note: Difficulties arise comparing refractive error prevalence across studies as differing dioptric cut-off criteria have been employed between studies to define each refractive error category. However, the increased implementation of the RESC protocol [25] addresses this, and in recent years the RESC protocol has come to be regarded as the gold standard. The definitions for refractive error classification used in the current study follow the RESC criteria apart from our added requirement of visual acuity to classify myopia. A further confounder when making prevalence comparisons derives from the use or not of a cycloplegic drug to inhibit subconscious activity of the focusing muscles within the eye. With cycloplegia, any latent hyperopia habitually compensated for by muscular effort, or any pseudomyopia existing due to muscle spasm, will become manifest in a relatively small percentage of children. In both conditions there is a plus-wards shift in the degree of the refractive error. Consideration as to whether the drug may cause the reverse to occur, a minus-wards shift (for which a mechanism is understood [85]), is rarely entertained despite some literature to support this occurring in up to 26% of normal children [86–88] and up to 85% of children with retinopathy of prematurity [89]. Either way, our decision to not use a cycloplegic may have therefore resulted in some children with high hyperopia being missed, but does not affect the private versus government school comparisons as all screenings followed the same protocol. Table 1 should be read with these factors in mind

Internationally the prevalence of myopia has been shown to have risen dramatically in Asia in recent decades and to be greater in the youth of urban communities compared to non-urban areas [13], suggesting that a greater prevalence of refractive errors and other visual conditions are likely to be found in Istanbul, the largest city of Turkey, than in the rural areas that have previously been examined. This expectation is also enhanced by considerations of prevalence of visual anomalies in children from suburbs of lower socioeconomic status, who would be expected to receive reduced health and educational experiences or opportunities. In particular, the concern with monitoring the prevalence of myopia derives from the potential for visual impairment or blindness that accompanies the higher degrees of myopia [14].

In an earlier study we have demonstrated that the refractive distribution for children of Middle Eastern background but residing and being educated in Australia, is similar to the known Australian norms for Caucasians [15]. Such results implicate educational style, especially years of schooling and associated near work (see review [13]), as significant environmental influences in the development of refractive error [16, 17]. Other research implicates factors such as time outdoors [18–20], birth weight [21], higher paternal occupational social class [22] and higher level of education [23] as influences on the induction and progress of myopia, suggesting that pressures for achievement may affect refractive distribution. Hence, it was considered imperative that baseline studies be carried out in a large metropolitan city in Turkey, such as Istanbul at the gateway between Asia and Europe. Apart from informing public health policy, longitudinal observations will also facilitate a wider understanding of the environmental drivers that operate in the genesis of myopia.

Thus, the principal aim of the current project was to conduct a preliminary school screening to gain insight into the visual status and the prevalence of myopia and other ocular conditions in school populations covering the extremes of the range of socioeconomic conditions in Istanbul. Ethnically similar school populations from nearby suburbs near central Istanbul were chosen given that they share the same national curriculum and geographic environment (e.g. the same hours of sunlight), but come from different socioeconomic clienteles. In the Istanbul of today, anecdotal evidence indicates that children attending private schools usually start school at an earlier age than those in Government schools, have greater access to good nutrition, less cramped housing, better health care, better access to books and a more intense schooling including a requirement for a laptop computer at school. Furthermore, from kindergarten on, children at private schools are expected to participate in several hours of organized sport and outdoor activities per week. By comparison public authorities in Turkey do not offer generalized preschool education [24], government schools do not begin organised sport programs until Year 3, and prior to 2010 attending children had little if any access to personal computers.

This project has resulted in the first data from Istanbul on the number of children requiring referral for further clinical investigation based on the prevalence of refractive errors, suspected amblyopia and strabismus.

## Methods

The parents of a total of 1014 students across three nearby schools in central Istanbul, two privately funded and one publicly funded, were invited to participate in the study to be carried out during school hours. Approval for the study was obtained from Istanbul Surgical Hospital’s Human Research Ethics Committee, the Turkish Department of Education and the Turkish Ministry of Health. The study adhered to the tenets of the Declaration of Helsinki. Written informed consent was required from at least one parent of each child and the verbal assent of all children was obtained before examination.

This study was intended as a school screening and did not conform fully to the standardised Refractive Error Study in Children (RESC) epidemiological protocol now adopted in a number of regions of the world [25]. First, the school populations were selected for socioeconomic diversity within a certain locale, rather than randomly sampled. Second, cycloplegia was not used at the behest of the Principals of the schools involved, and thus the data gathered was restricted to that associated with refractive errors manifest at the time of presentation. This issue is considered further in the Data Analysis section and in the Discussion.

In order to understand the visual status of Turkish city-dwelling children from both ends of the socio-economic extremes, government and private schools in the municipality of Beşiktaş in central historic Istanbul were contacted. During a scheduled but informal interview with the school principal to explain the project objectives and procedures, the general socioeconomic profile of the parents of children attending the schools was ascertained in terms of SES. One government school was deemed to be in the lowest SES group (i.e. with parent wages in the lowest 5% of household income averages for Istanbul) [26]. This school catered for children aged 4 to 10 years (grades Kindergarten to Year 5) and charged no fees. Two nearby privately-run schools that catered for children aged 3 to 10 years (grades Preschool to Year 5) were selected for comparison and to provide an age-extension into the preschool age group. Parents with children attending these two schools were deemed in the top level of SES (i.e. in the top 5% of household income averages for Istanbul) and the schools charged fees in the top bracket of private schools in Turkey.

Parents were requested to complete a questionnaire (see ‘Additional file 1’) relating to the time of their child’s last vision assessment (‘never’, ‘over 2 years ago’, ‘between 1 and 2 years ago’, ‘within the last year’) and to indicate any concerns or other comments. Each parent from the government school completed this section concerning last eye examination, however 2.5% of parents at the private schools failed to supply this information.

Vision screenings were performed in 2009 by trained personnel who included a paediatric ophthalmologist (FS), an orthoptist (SA) and an ophthalmic nurse. The same protocol was used at each school between February 2009 and June 2009.

The examination process began with presenting (habitual) distance visual acuity, with or without spectacles as appropriate, using a computerised EDTRS LogMAR Chart (Nidek SC-2000 chart, NIDEK Co., LTD) displaying letters or Lea symbols as appropriate to the child’s reading ability. Visual acuity testing was discontinued when, despite encouragement, the child misidentified more than half the line, or once 6/6 (LogMAR 0) was achieved. Cover test (unilateral and alternating) was then performed and children with strabismus were noted. To ascertain the suspected presence of amblyopia, not only was a visual acuity difference between the 2 eyes of at least 2 lines required, but also the cover test results, the child’s autorefractor data (see below) and their status with respect to wearing spectacles, as well as the time since their last eye examination were each considered. In particular, a child was not considered as an amblyopia suspect if poor acuity was found and the autorefraction findings at the same time indicated potential for spectacle correction to improve vision. In the absence of a misalignment of the visual axes of the eyes and/or a refractive reason for reduced acuity, organic reasons for reduced visual acuity beyond those detectable with an ophthalmoscope were not pursued as part of this screening and these children were referred.

Autorefraction was undertaken using a Nidek ARK-530A (NIDEK Co., LTD) autorefractor/keratometer (with automatic fogging activated to minimise potential accommodation). Spectacles were not worn during this procedure. Ten readings were taken and averaged. Autorefraction measurements of sphere and cylinder were converted into spherical equivalent refraction (SER), where SER = Sphere + (Cylinder/2).

Refractive categorization was defined as follows. The presence of myopia utilized the customary dioptric cut-off for myopia of at least −0.50D SER, but was superimposed with a criterion that unaided vision should be 6/9.5 (LogMAR 0.2) or poorer to exclude the possibility of instrument myopia whilst using the Nidek ‘closed box’ style of autorefractor [27]. This procedure has been reported to be reliably similar to a subjective determination of the need for spectacles in subjects of similar age, even when cycloplegics are not used [27]. Furthermore, recent studies have demonstrated that the use of an autorefractor even without cycloplegia results in a sensitivity of over 0.90 to detect moderate myopia and moderate hyperopia [28, 29]. Hyperopia was defined as SER ≥ +2.00D and astigmatism as ≥1.00 DC.

Colour vision was tested using the Ishihara test. Gross stereopsis was assessed using the Standard Titmus Fly test (Bernell, USA).

Parents were advised of the outcome of the screening by letter and advised to seek further ophthalmic assessment for their child if any of the following had been found: presenting visual acuity in either eye poorer than 6/7.5 (LogMAR 0.1) or a difference in acuity of two lines or more between eyes, hyperopia greater than +1.00 D SER, myopia greater than −1.00 SER when accompanied by presenting visual acuity poorer than 6/7.5, astigmatism greater than 0.75 DC or anisometropia greater than 1.00 DS, an inability to identify at least two of Ishihara plates and/or not being able to trace one of the coloured pathway plates, an inability to see the Titmus Fly in 3-D (800 s of arc disparity). Parents of the children at the government school were offered free eye care and spectacles at a private ophthalmological clinic if they were unable to afford eye care elsewhere.

### Data analysis

Children with suspected amblyopia (*n* = 15) were not included in the refractive status statistical analyses as a significant difference in refractive error between the amblyopic and the fixating eye would be expected [30]. Data from children with suspected organic causes for their reduced acuity (*n* = 4) were also not included in refractive analyses.

Descriptive statistical analysis was carried out using SPSS (v20). Means and standard error of the mean (SEM) are presented. Right and left eye SERs were highly correlated for the children not suspected of amblyopia (*r* = .80, *p* < .001), therefore all statistical analyses relating to refractive error were performed only on right eye data. Two 2(school type: private and government) × 3(SER refractive group: myopia, emmetropia and hyperopia) ANOVAs were carried out to compare refractive groups for differences in age between the different types of school (note that as expected, refractive error was skewed for the myopia and hyperopia groups, but not the emmetropia group, thus non-parametric statistics were used to confirm all significant outcomes). The differences in refractive error between schools were compared using the non-parametric Mann-Whitney U test. The level of significance attributed to failure to meet the criteria for the other visual variables as shown in Table 2 was established through the use of Student t-tests for continuous variables and χ^2^ tests for categorical variables.

**Table 2:** Summary of the differences in prevalence between children attending a government school versus a private school for the visual characteristics assessed

	Criterion	Government school *N* = 227	Private school *N* = 596	Significance
Visual acuity @6 m	6/6 or better in both eyes	76.2%	86.4%	0.002
	Visual acuity ≤ than 6/9.5 in better eye	7.0%	2.5%	0.034
	Visual acuity ≤ than 6/19 in better eye	6.2%	0.5%	0.017
Spherical equivalent refraction	≥ + 2.00 DS	0.6	0.6	NS
	< −0.50 DS and VA ≤ 6/9	6.90%	5.60%	NS
Astigmatism	≥1.00 < 2.00 DC	5.10%	4.40%	NS
	≥2.00 DC	1.90%	2.90%	NS
Anisometropia	≥ 1.00	5.60%	4.90%	NS
Amblyopia/strabismus	See Methods	4.80%	1.00%	0.048
Last vision assessment	None	64.8%	22.4%	<0.001
Presented wearing spectacles	Yes	5.7%	8.7%	NS
Colour vision	Missed ≥2 plates	0.9%	4.9%	0.040
Stereopsis	> 800 s arc	4.85%	0.17%	0.002
Referred	No spectacles and VA < 6/7.5	13.7%	5.4%	0.004
	Current spectacles not giving 6/6	1.3%	1.8%	NS
	Presenting acuity ≥6/7.5 but autorefractor ≥ + 1.00DS	1.3%	1.0%	NS

## Results

**Fig. 1:**
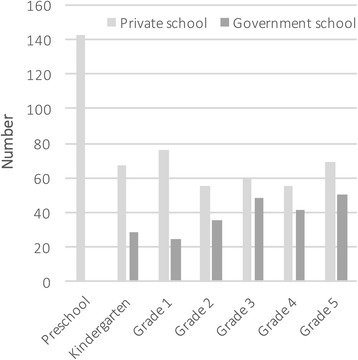
Number of children in each grade according to type of school

Eight hundred and twenty-three students out of a potential 1014 students in the three schools (81.2%) participated, with almost identical participation at both the government and private schools (81.1% and 81.3% respectively). Of those screened (see Fig. 1), 596 came from the private schools (50.0% male) and 227 from the government school (51.3% male). The mean age for the total population was 6.7 ± 2.2 years, range 4 to 10 years (6.6 ± 2.1 years for males and 6.8 ± 2.2 for females). At the government school the mean age was 7.7 ± 1.9 years and for the corresponding grades at the private schools it was 7.1 ± 1.9 years. This difference stemmed largely from a higher proportion of children in the early school grades at the private schools (28.6%) compared to those at the government school (12.3%) (see Fig. 1). The mean age of children across all private school classes, including the pre-school classes, was 6.3 ± 2.1 years. Not surprisingly the two-way ANOVA for age showed a significant main effect for school, F(1791) = 13.94, *p* < .001. A summary of the relative prevalences according to type of school for the visual characteristics described below is given in Table 2.

### Presenting distance visual acuity

Mean visual acuity across all eyes (*n* = 1646) was 6/6.6 [LogMAR 0] (notably, this value is subject to the ceiling effect imposed by limiting testing to the 6/6 line of letters). Of all 823 children, visual acuity was 6/9.5 [LogMAR 0.2] or better in 94.2% of children using either letters or shapes as appropriate, 6/7.5 [LogMAR 0.1] or better in 88.2%, and 6/6 [LogMAR 0] or better in 83.6%. At the government school only 76.2% of children were able to read 6/6 [LogMAR 0] or better with each eye, whereas at the private school this figure was 86.4%, which is a significantly (*p* = .0015) greater proportion than for the government school. For those with acuity poorer than 6/7.5 [LogMAR 0.1] in each eye, 3.8% of all children (*n* = 31) were limited to identifying just the 6/9.5 line [LogMAR 0.2] with their better eye (7.0% and 2.5% at the government and private schools respectively). A further 2.1% of all children (*n* = 17) were considered to be currently functioning as ‘visually impaired’ as they were limited to identifying only the 6/19 line of letters [LogMAR 0.4] with their better eye (6.2% and 0.5% for government and private schools respectively). No child was measured as having distance visual acuity worse than 6/24 [LogMAR 0.6] with their better eye.

### Refractive error

Fifteen children were considered as amblyopia suspects and four other children were considered more likely to have an organic reason for the reduced vision in one eye based on the similar emmetropic refractive errors for both eyes and the negative results from the cover test. Hence, the results of these 19 children were not included in the analyses of refractive error prevalence.

For the remaining 804 (97.7%), the mean right eye SER by non-cycloplegic autorefraction was −0.16 ± 0.70DS. This did not differ significantly between schools. Of these 804 children, 6.0% (6.9% in the government school and 5.6% in the private schools) were myopic using both autorefractor and unaided distance acuity criteria. As expected with increasing age, the prevalence of myopia in the private schools rose from 2.8% for those in Pre-school or Grade 1 to 16.1% for those in Grades 4/5. Similarly, in the government school myopia prevalence rose from 3.8% in Grade 1 to 16.5% for Grades 4/5. Only 0.6% demonstrated an SER of at least +2.00D (almost equal proportions at both government and private schools) and are therefore clearly hyperopic. A further 2.6% had an SER > +1.00D but <+2.00D which is suggestive of considerable hyperopia given the style of autorefraction employed. Thirty-seven children (4.6%) were found to have astigmatism of at least 1D but less than 2D (5.1% in the government school and 4.4% in the private schools), and a further 2.6% had astigmatism of at least 2D (1.9% in the government school and 2.9% in the private school). With respect to anisometropia (≥1.00D difference in SER between right and left eyes), 5.1% had anisometropia of at least 1D but less than 2D (5.6% in the government school and 4.9% in the private schools), and a further 1.1% had at least 2D of anisometropia. No significant differences were noted between males and females.

### Suspected amblyopia and strabismus

Of the total 823 children screened, 1.5% were found to be strabismic on the cover test and 1.8% showed signs of amblyopia, i.e. at least two lines of uncorrectable difference in visual acuity between the two eyes and either a hyperopic difference of at least 2.00DS in the eye with poorer acuity (*n* = 2), or uncorrected astigmatism of at least 1 DC in the eye(s) with poorer acuity (*n* = 13). Six came from the private school (1.0% of 596 children) whereas nine came from the government school (4.8% of 227 children) which represent significantly different proportions (χ^2^ = 3.90 df = 1 *p* = .05).

The mean distance visual acuity of all eyes with potential amblyopia (irrespective of whether it was the right or left eye that was amblyopic) was 6/25.5 ± 12.5 [LogMAR 0.62] (range 6/9.5 [LogMAR 0.2] to 6/48 [LogMAR 0.9]) whereas the mean acuity for their non-amblyopic eyes was 6/7.4 ± 1.9 [LogMAR 0.1] (range 6/7.5 [LogMAR 0.1] to 6/9.5 [LogMAR 0.2]). The mean SER of all amblyopic eyes was +0.01 ± 1.23D (range − 1.87 to +3.13D) and fellow non-amblyopic eyes −0.35 ± 0.57D (range to −1.75 to +0.25 D).

### Last vision assessment

**Fig. 2:**
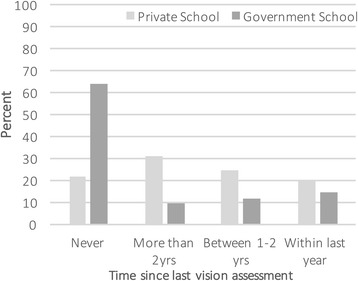
Time since last vision assessment according to type of school, presented as the percentage of students within each school type

One third of all parents reported that their child had not previously attended a visual assessment (64.8% of the government school, and 22.4% of the private school children). The difference in non-attendance for eye care according to type of school is highly significant (χ^2^ = 44.40 df = 1 *p* < .001). The remaining two-thirds of the children had attended an eye examination within a time ranging from in excess of two years ago to within the last year (of whom, two-thirds attended the private schools (see Fig. 2)).

### Spectacle wear

Of the total population (including suspected amblyopes and those with suspected organic causes for reduced vision) only 7.9% were wearing spectacles at the time of the screening (8.7% of private school children and 5.7% of government school children, which is not a significant difference). The autorefractor classification of these children was as follows: 3.5% of all children were myopic ≤ − 0.50D, 1.0% had low hyperopia between +1.00D to +1.99D, 0.4% had moderate to high hyperopia ≥ + 2.00D. An astigmatic component ≥1.00 DC but <2.00 DC was found in the autorefraction of 1.2% and a further 1.8% had an astigmatic component >2.00 DC. Only 2 of the 15 amblyopes (both moderate myopes with significant astigmatism) were wearing spectacles.

Of the children wearing spectacles, 76.9% achieved better than 6/7.5 [LogMAR 0.1] visual acuity (80.8% in the private schools and 61.5% in the government school). Of the remaining 15 spectacle wearers with lower than expected acuity, mean acuity was 6/8.7 ± 1.18 [LogMAR 0.16] of whom 12 were myopes who could reasonably be expected to have shown mild progression with their myopia since the last eye care visit.

### Colour vision and stereopsis

A total of 3.8% of all students were unable to identify at least two plates in the Ishihara colour vision test: two children from the government school (0.9% of the 227) and the remaining 29 from the private schools (4.9% of 596). This higher prevalence of colour vision impairment in the private schools was significant (χ^2^ = 4.2 df = 1 *p* < .04). A total of 1.5% of all children were found to have stereopsis poorer than 800 s of arc (11 from the government school and only 1 from the private schools, which was significantly different (χ^2^ = 9.8 df = 1 *p* < .002). Two of these children were amblyopes and another two had reduced vision deemed to be organic in origin.

### Referral

Eighty-six children (10.4% of all children assessed) required referral either because (i) they did not own spectacles and their presenting acuity was inadequate (13.7% of children attending the government school, 5.4% the private school), or, their presenting acuity was 6/7.5 or better but the autorefractor indicated hyperopic refractive error ≥ +1.00DS (1.3% and 1.0% at the government and private schools respectively), or, (ii) their current spectacles did not provide an expected 6/6 visual acuity (1.3% and 1.8% at the government and private schools respectively). Thus, only 8.2% of children from the private school were deemed in need of further assessment whereas 16.3% of children came from the government school. The difference in the proportions of children ‘not owning spectacles and also having inadequate visual acuity’ varied significantly between type of school (χ^2^ = 8.50 df = 1 *p* < .01).

## Discussion

This is the first report on the visual status of children in Istanbul and indicates a need for government sponsored screening programs, especially for children from lower socioeconomic backgrounds. As expected, the findings indicate that children from more privileged families are more likely to have already received eye care, more likely to experience better visual acuity regardless of whether spectacles are available to them or not, and less likely to exhibit amblyopic signs. With one in ten of children in the entire sample requiring immediate attention for relatively easily remedied visual problems, and two-thirds of these being children from a low socioeconomic background, this study underscores the imperative for vision tests to be included in community- or school-based public health screenings.

### The need for eye care

The pre-eminent issues relating to eye care concern strabismus, amblyopia and refractive errors as they are the most common vision-based afflictions in children and are usually manageable such that visual acuity and visual comfort can be improved to minimise any impact on learning and lifestyle into adulthood [31–34]. To date, only a small number of studies on the visual status of children have been carried out in Turkey and are reviewed and summarised in Table 1 [9–12, 35, 36].

Strabismus and amblyopia. Strabismus often leads to amblyopia and can be cosmetically displeasing which leads to social isolation, but can be surgically managed and the long-term impact on the child decreased [31, 34, 37, 38]. The current finding of 1.5% of children observed to have strabismus is at the lower end of the range previously found in Turkey, and is at the lower end of the range of prevalence studies in the Middle East, European and Asian populations [37–41]. Notably however, in the current Istanbul study, the prevalence of amblyopia was found to be 3.9 times greater in children from the government school than children from the private schools. It cannot be ruled out that amblyopia may have been present at an earlier age in the private school children, but that the higher incomes of parents of higher socioeconomic status facilitated earlier identification and effective treatment. Poverty has been reported in the US as a factor in poor amblyopia treatment outcomes, as access to interventions is significantly restricted by their cost [42–44]. Additionally, Unsal et al. [12] found that the children of working women in Turkey were significantly more likely to present to a school screening with visual impairment, suggesting that the mother did not have time to take their child for eye care, or, there was insufficient family income to pay for eye care services.

Refractive error prevalence. In keeping with worldwide studies, uncorrected refractive error affects a far greater number of children in Turkey than does strabismus or amblyopia as demonstrated here and in the other Turkish studies (see Table 1). Notably, Gursoy et al. [10] considered their finding of a comparatively high prevalence of myopia in a rural university-based city may derive from incomplete cycloplegia leaving some residual focussing capability (see ‘Note’ below Table 1), even though the RESC protocol for drug administration and refractive assessment was utilized.

Myopia is usually the first refractive error category to be considered given that the eye is at far greater risk of significant age-related ocular morbidity according to WHO standards [45], and likely to lead to low vision or blindness. In East Asia, a doubling of the numbers of children with myopia, particularly higher degrees of myopia has occurred every decade over the last 30 years which precludes genetics as being the main determinant of whether a child develops myopia and thus implicates environmental and lifestyle factors [13, 46–48]. In Shanghai, 19.5% of university students have high myopia greater than −6.00DS and are at high risk of pathological consequences that will significantly affect quality of life and employment [45]. One may argue that Turkey does not have significant numbers of persons with myopia. Indeed, it did appear that Australia was not experiencing the shift towards higher numbers of myopic children in the early 2000’s [49] at a time when Asian countries had already noted this. However, a decade later it is apparent that the prevalence of myopia in Australian children may be rising [50]. Although it has been long held that factors such as parental education and hours of nearwork can be drivers for the development of myopia, more subtle aspects such as urban/rural living and the number of hours of outdoor activities have also been noted as important [13, 20, 48]. Thus, we consider it important that well-designed baseline studies be undertaken in Istanbul to document the prevalence of myopia as a means to inform and monitor the need for public health initiatives, and also aim to better understand the many parameters driving the genesis of myopia.

Our finding that 6.0% of children (mean age 6.7 years) have myopia is low compared with East Asia, but not lower than that generally found in Caucasian countries for children of similar age. Even lower prevalences of myopia were found in rural Turkey where farming is common and attendance at the government school is generally just half a day [9, 11]. On the other hand, a much higher prevalence has been found in one Turkish rural centre that is also a large university town [10]. Considering just the older children in the current study, 16% of the 9–10 year olds were myopic, which is in keeping with our other published data of a prevalence of 17.2% for children aged 10–11 years of predominantly Lebanese background but residing in Australia and undertaking the standard Australian school curriculum [15]. The only published study concerning myopia prevalence in adults in Turkey looked specifically at medical students in 2007 [51] and found 32.9% had at least −0.75D myopia. Although seemingly high, this finding for Turkish medical students is low compared to the prevalence of over 90% found in east Asian medical students (using a − 0.50D cut-off) [51]. Thus, it is important when the results of vision screenings are reported that a thorough description of the demographics of the subjects are also provided so as to aid understanding of the likely genetic predisposition and potential environmental triggers for myopigenesis.

The similarity in the prevalence of myopia between the two different types of school in the current study was unexpected. However, all schools undertake the same academic curriculum set by government and the children from the three schools have very similar ethnic backgrounds and physical locations even though they come from SES lifestyle environment extremes. The size of dwelling may also be similar, as most of the private school children apparently live in new apartment blocks in inner Istanbul. On the other hand, parental myopia and higher education are well-known risk factors and the parents from the private schools pay very high school fees necessitating a higher socioeconomic level, derived in many cases as a result of a higher educational background. The advantage of higher income has long been known worldwide to be accompanied by an increasing prevalence of myopia [52–57] and has this been noted in rural centres in Turkey [9]. Therefore, one might argue there is a greater likelihood of myopia among the private school parents compared to parents of children attending low-income government schools. Notably, in Turkey only 12% of those over the age of 15 years have tertiary education [58]. However, this phenomenon with respect to higher parental income was not apparent in the current study, even when taking into account the dissimilar age profiles between our schools (6.7% and 6.9% for myopic children of comparable age in the private and government schools respectively). An association was also not seen in urban Iran when using father’s education as the variable [59]. Unfortunately, the refractive status and education level of parents in the current study was not available, both of which represent limitations to interpretation of our data. Counter to these arguments, the private schools in the current study have an extensive outdoor sport program that starts earlier than the government school’s program, which may according to recent research [20], be an ameliorating factor in the potential early onset of myopia for the private school children.

Hyperopia has traditionally received less attention than myopia as, from a medical view-point, hyperopia is seldom a risk factor for ocular complications [60]. However, hyperopia is increasingly understood as being associated with asthenopia and less successful educational outcomes, and thus should receive greater public health intervention [60–62], particularly for hyperopia that has limited or no impact on visual acuity [63]. The current non-cycloplegic findings of 0.6% children having moderate hyperopia (> + 2.00D) and 4.1% having milder hyperopia (> + 0.75, <0.2.00D) are somewhat lower proportions than typically found in populations of lower socioeconomic background or rural location, where low to moderate hyperopia is common [17, 38, 48, 54, 64, 65]. The use of a ‘closed box’ style autorefractor in the current study (i.e. where the child looks at a picture inside the shoe-box sized instrument positioned in front of their face, rather than looking through a semi-silvered mirror sitting on top of the instrument to a distant object across the room), may have limited the detection of children with hyperopia and somewhat minimised the degree of hyperopia detected, in that the closed-box can promote awareness of nearness and in turn inhibit the degree of hyperopia manifested [66].

Medically, those with astigmatism are not at risk of developing ocular complications, but may experience significant asthenopia (eyestrain, headaches, blurred vision) [61, 67, 68] and thus impaired learning. Few Turkish studies have addressed astigmatism, however our finding of 12.9% is similar to the only other Turkish study using a reliable autorefractor cut-off criterion for astigmatism [69].

In most countries neighbouring Turkey, there is limited data (particularly in the English language literature) relating to the prevalence of refractive errors in children (see Table 1). However, in Iran there have been six significant cycloplegic refractive errors studies in the last five years (all adopting the RESC procedures with large sample size and in large cities or towns of population at least 200,000 people) [59, 70–74]. All yielded a prevalence of myopia (≤ − 0.50D) under 5% in children of similar age to those in the current study. The prevalence of hyperopia ≥ + 2.00D under cycloplegia has been described as ranging from 7% to 20.9% in 5–15 year olds in Iran [59, 70–74] and is far greater than that found in our study. Two Iranian studies additionally examined pre-cycloplegic data and found that, as would be expected, a far greater number of children (56% [72] to 76% [75]) fell in the category of low hyperopia (≥ + 0.50D but <2.00D) in the non-cyclopleged state than during cycloplegia (19% to 33% respectively).

What is apparent from all these figures is that our refractive data for central Istanbul is generally consistent with these recent trends showing a higher prevalence of myopia in urban versus truly rural towns. In modern urban areas there has been a shift in society towards higher education standards for children, more urban white-collar work and less outdoor play and employment. Further evidence that argues strongly for an important role for environmental influences relating to location comes from studies examining the change in prevalence with immigration to a country of higher prevalence resulting in ethnic-related increase in prevalence [15, 76, 77] or conversely, immigration to a country of lower prevalence resulting in ethnic-related decrease in prevalence [76]. Although ethnicity was not pursued in the current study, it would be of value in future larger investigations.

### Previous eye care as a public health issue

The issue of whether children in Turkey and neighbouring countries have previously received eye care has been addressed by several groups (see Table 1). Our data reveals a stark contrast between the 77.6% of children from a higher socioeconomic background living in Istanbul who have previously had an eye examination versus the less than half this proportion (35.2%) for nearby government school children who are generally of lower socioeconomic background. Other Turkish studies do not address previously received eye care, although in Iran it appears that health checks are obligatory for all children before enrolling in school [71]. Indeed, Jamali et al. [71] found that 85.3% of children had completed the preschool amblyopia screening, and furthermore, that children who were compliant wearing their spectacles were half as likely to develop amblyopia.

An indirect indication of previous eye care comes from looking at the number of children presenting with spectacles. In the current study (where children are of relatively younger age and thus less likely to be myopic), only 7.9% presented wearing spectacles. However, only 5.7% of children from the predominantly lower socioeconomic background government school presented wearing spectacles compared with 10.6% of children from the private school. This again highlights the advantage of a higher socioeconomic background, given that the prevalence of myopia was similar in both the private and government schools.

### The value of public health screening to detect those requiring eye care

The issue of how many children during a screening will be identified to require further care is of utmost importance to public health policy makers, but does depend on the scope of the screening, the age of the children, the locale, etc. The current study has also highlighted socioeconomic status as an important factor, with twice the number of children (16.3%) in the lower socioeconomic background school requiring referral compared to those in private schools (8.2%).

Measurement of vision/visual acuity is a useful screening tool (see Table 1), as presenting acuity during a screener can reliably detect refractive error [78, 79]. Presenting distance vision is particularly useful to detect myopia, but to ascertain the presence of all three refractive errors the combination of both distance and near vision testing gives best results [78]. However, perhaps the best indicators to develop public health policy regarding the efficacy of screening children comes through comparison of the relative proportions of children with poor vision under different conditions: (i) when uncorrected (as this identifies the total number of children affected), as against (ii) habitual vision (which, with the first item, then identifies the resourcefulness within the community to provide any necessary optical aids), and finally (iii) best corrected vision (which when considered against the first item indicates the proportion requiring spectacle correction, as well as the small proportion of children who despite best correction will always be dependent on community support to adequately survive because their vision is still quite inadequate). Thus, the cost of public health screenings should not be considered simply in monetary terms, but also against the personal burden for those experiencing visual anomalies. For example, Caca et al. [9] showed that 13.9% of children could be raised from a status of ‘visually impaired’ to ‘normal or near-normal vision’ simply through correction of their refractive error. Furthermore, they showed that 4.1% of all children changed their designation from ‘functionally blind’ to ‘useful’ if not ‘normal’ vision simply with spectacles.

Poor best-corrected visual acuity has been found to be most likely associated with strabismus, hyperopia, astigmatism and anisometropia by Gursoy et al. [10], hence the importance of including visual acuity status and refractive error determination in the one vision screening (particularly if children are subsequently spectacle-compliant [71]). However, in the current study there was notably a significant difference between the proportions of government school children (6.2%) and private school children (0.5%) experiencing ‘visual impairment’ as their habitual state at school (i.e. poorer than 6/19 line of letters with their better eye). By WHO standards, this level of impairment affects quality of life and ability to hold employment, and underscores potential socioeconomic contributing factors [80].

The current study was initiated as a pilot to obtain a public health perspective on oculo-visual status of children in Turkey’s largest city. The lack of refractive data on 19% of non-participants at each school may be perceived as an equally distributed overall bias. However, because the relative proportions of children identified with anomalies at each type of school were not equal, bias from non-participation although numerically equal may not actually be the same. It could be argued that those who did not participate had already been identified as having a visual anomaly, in which case our prevalence findings may represent an overall underestimate. Some non-participation was reported by teachers to be simply due to illness on the day of screening. The reliance of non-cycloplegic data in the current study has been mentioned and is further discussed at the foot of Table 1 (see^ a^). This aspect suggests also that our prevalence findings for hyperopia will be an underestimate.

Future studies of visual status of children in Turkey should use appropriate randomised cluster sampling stratified by socioeconomic status and aim to determine what true differences in schooling exist for children of differing SES covered by the study, the socioeconomic status of the family, parental education and income, and importantly, parental refractive error and compliance of children already prescribed spectacles. Whilst demographic information is customarily gathered through a questionnaire to parents, it must be recognised that educational attainment is generally poor in Turkey (prior to 1997 compulsory schooling comprised only 5 years and thereafter increased to 8 years, and in 2007 literacy for persons aged 15 and over stood at only 78.5% for females and 94.4% for males [81]).

## Conclusion

In summary, this study is the first to detail the prevalence of refractive error, amblyopia and strabismus in children from the extremes of socioeconomic status in inner Istanbul. It is suggested that at least one in ten children requires ongoing visual care. Children receiving education at a government-run school (and likely to represent the lowest socioeconomic sector of Istanbul), were three times less likely to have had their eyes examined previously, exhibited poorer habitual visual acuity and presented with a higher prevalence of amblyopia, but had a similar prevalence of myopia as for children from the private schools. Hence, given the relatively high numbers of young people in Turkey and the predominance of children in lower socioeconomic circumstances, a plan for eye care as part of standard public health care delivery is indicated. Further, developing a scientific understanding of the factors involved in the prevalence of refractive error in Turkey will be particularly useful to understanding potential ameliorating agents should the prevalence of myopia rise in Turkey with time, as has occurred in countries where striving for academic achievement has significantly increased demands for extended near work and shifted lifestyles. Thus, coping with the increase in myopia prevalence is not just a public health issue, but also an important social issue.

## Additional file

Additional file 1: Parent Questionnaire. The questionnaire is to be completed by a parent or guardian of each child participating in the school-based vision screening, and covers the time of the child’s last vision assessment and whether there are any concerns or other comments about the child’s eyes and vision. (DOCX 66 kb)

## Abbreviations

DC: Dioptres cylinder
DS: Dioptres sphere
FS: Funda Şerefhan (Author)
RESC: Refractive Error Study in Children
SA: Serap Azizoğlu (Author)
SER: spherical equivalent refraction
SES: socioeconomic status
WHO: World Health Organisation
