# Supplementary material for: Evidence for the need for vision screening of school children in Turkey
Source: BMC Ophthalmol. 2017 Dec 2;17:230. doi: 10.1186/s12886-017-0618-9 (PMC5712108; doi:10.1186/s12886-017-0618-9)
Supplement: Additional file 1: — Parent Questionnaire. The questionnaire is to be completed by a parent or guardian of each child participating in the school-based vision screening, and covers the time of the child’s last vision assessment and whether there are any concerns or other comments about the child’s eyes and vision. (DOCX 66 kb) [file 12886_2017_618_MOESM1_ESM.docx]

Dear Parent/Guardian,

Istanbul Surgery Hospital will be carrying out a Vision Screening program at your school. If you agree to your child being involved in this program, please answer the questions below, then sign and return this form to the school. Results of the screening will be sent to you.

When was your child’s last vision assessment? (tick one box)

☐ Within last year

☐ Between 1 to 2 years ago

☐ Over 2 years ago

☐ Never

Do you have any concerns or comments related to your child eyes or vision?

____________________________________________________________________________________________________________________________________________________________________________________________________________________________________________________________________________________

Your child’s name _____________________________________

☐ Male ☐ Female

Date of birth __________________________

Class________

I, parent/guardian (Name)____________________________ give permission for (print child’s name) ___________________________ to be involved with the school vision screening program.

Parent signature _____________________

Date __________________

Thank you for your participation
